# Teaching Careers: Exploring Links Between Well-Being, Burnout, Self-Efficacy and Praxis Shock

**DOI:** 10.3389/fpsyg.2019.02255

**Published:** 2020-02-18

**Authors:** Julie Ballantyne, James Retell

**Affiliations:** The University of Queensland, Brisbane, QLD, Australia

**Keywords:** teacher education, music teacher attrition, professional identities, pre-service and in-service education and training, occupational stress

## Abstract

Burnout and attrition are issues facing many professions. In a bid to better understand this phenomenon and ways to address it, this paper explores experiences of praxis shock, well-being, burnout, and self-efficacy during teachers’ careers. Regression and mediation analyses of 836 responses to a questionnaire reveal that praxis shock may occur at multiple points in a music teachers’ career. Findings reveal that praxis shock predicts patterns of reported burnout, well-being and self-efficacy. This impacts on the development of productive professional identities, career satisfaction and success. Evidence is presented regarding praxis shock and its impact across a teaching career.

## Introduction

Professional identities are the ways teachers make sense of themselves within their professional lives. They encompass and are interrelated with personal identities, social and cultural identities and norms, and professional roles and contexts. Research consistently notes the centrality of teachers’ professional identities to all aspects of their job. Professional identities particularly impact on teachers’ abilities to navigate the complexities of ‘being a teacher’ (Beijaard et al., 2004; Sachs, 2005). This is because the notion of ‘who I am as a teacher’ influences, and is influenced by, self-perceptions of agency, self-efficacy, effectiveness, and job satisfaction (Day and Kington, 2008; Skaalvik and Skaalvik, 2014). Although many inroads have been made into researching professional identities and the experiences of beginning teachers (see for example Flores and Day, 2006; Beauchamp and Thomas, 2009; Hong, 2010; Pillen et al., 2013), there is still a need to better understand how teachers traverse through other career periods. This article explores how the components of perceived professional identity (including praxis shock, burnout, well-being, and self-efficacy) interact across the teaching career.

Research on the career and identities intersect where both fields acknowledge that broader issues in a person’s job and life are relevant when exploring changes over time (Day and Gu, 2007). As people progress through their chosen profession, their experiences and the ways that they respond to all aspects of their work, change and develop. Research suggests that career development is seen as a “whole-life” endeavor, necessarily encompassing the myriad facets of a person’s life that may impact on their career (Litano and Major, 2016). Individuals who are in the profession of teaching are therefore expected to experience changes that will influence their career, over time.

Gu and Day (2007) emphasize that teachers’ abilities to “cope” in their professional lives are impacted by their identities and professional life phases, which are in turn mediated by issues in their personal and situated lives, their professional values, beliefs, and external policy agendas. The “ability to cope”, or remain resilient in the face of considerable job uncertainty, job stress, policy changes and career challenges, appears to be most challenging to teachers at the beginning of their careers, when they tend to burn out and leave the profession (Schaefer, 2013). Teachers’ responses to career changes and stress vary, but it does seem that teachers are leaving the profession because they are “burning out” (Parliament of Australia Media Release, 2019). As indicated by its name, “burning out” is often the end point of a negative response to work stress and emotional exhaustion (Skaalvik and Skaalvik, 2007). Burnout (and attrition) is affecting the profession (perhaps across the career span), and this is of grave concern to policy-makers, teacher education providers, employers and government bodies alike.

Research on attrition and burnout in teaching necessarily reflects the highly complex and multi-faceted experience of teachers in the workforce (Lindqvist et al., 2014). Burnout has been associated with mental and physical health (Hultell et al., 2013), as well as work environment and motivational factors (Fernet et al., 2012). It can also be attributed to ‘clients’ or students (Kristensen et al., 2005). Thus, when investigating teacher professional identities, measuring burnout provides a picture of the degree to which teachers feel fatigued as a direct result of their work, and therefore the likelihood of their remaining a teacher. Interestingly, recent work by Hultell et al. (2013), suggests that burnout might not exclusively be the domain of the early career teacher, finding that during their first 3 years of employment, teachers were, on average, relatively healthy with moderately low, albeit increasing, levels of burnout (p. 84).

The day-to-day life of a school teacher often represents a fundamental divergence from the expectations and ideals that are held prior to entering the profession. The feeling of surprise, shock or disequilibrium resulting from this experience has been termed *praxis shock* and is most common in the early years of teaching (Kelchtermans and Ballet, 2002; Stokking et al., 2003; Ballantyne, 2007). The mismatch experienced when one’s expectations of professional life do not align with the realities experienced working within the profession, is likely to be accompanied by a sense of disillusionment and disappointment. Managing praxis shock compounds the burden faced by early career professionals and those who experience praxis shock are likely to be less effective in their work, and may be unable to provide their schools and communities with the best possible provision of education. The experience of praxis shock can result in burnout (Ballantyne, 2007), and is also associated with attrition and reduced job satisfaction (Kelchtermans and Ballet, 2002; Ballantyne, 2005, 2007; Hong, 2010; Skaalvik and Skaalvik, 2010). Burnout can also be reduced or mitigated against “when teachers have a positive perception of their self-efficacy” (Betoret, 2006, p. 534). Arguably, the ways that teachers perceive praxis shock actually may be the key to successful identity development, and longevity in the career (Ballantyne and Zhukov, 2017).

Burnout, praxis shock and attrition are clearly interrelated areas of study examined by researchers in the fields of teacher education and teacher education (Kelchtermans and Ballet, 2002; Ballantyne, 2005, 2007; Hong, 2010; Skaalvik and Skaalvik, 2010). Burnout, praxis shock and attrition are also related to issues of job satisfaction and self-efficacy (Skaalvik and Skaalvik, 2010). Perceived self-efficacy is aligned with success in the profession, with Bandura (2006) maintaining that “the stronger the perceived self-efficacy, the higher the performance attainments” (p. 175). For the purposes of this study, the definition of self-efficacy was borrowed from Skaalvik and Skaalvik (2014), in that it refers to teachers’ beliefs about their own abilities to plan, organize, and carry out activities required to attain education goals” (p. 69). Skaalvik and Skaalvik found that better self-efficacy increased job satisfaction, and conversely, poor self-efficacy increased the likelihood of burnout in the profession. Previous research in this area links self-efficacy and psychological well-being, personal accomplishment, job satisfaction, and commitment (Zee and Koomen, 2016). Self-efficacy is strongly associated with images of self, and Beijaard et al. argue that such images “strongly determine the way teachers teach, the way they develop as teachers, and their attitudes toward educational changes” (2004, p. 108). It is important, therefore, when examining teacher professional identities over the career, to also investigate teacher self-efficacy.

Most studies in this area focus on the experiences of beginning teachers (Kelchtermans and Ballet, 2002, Kelchtermans and Ballet, 2002; Ballantyne, 2007; Hong, 2010; Shaw, 2016), and very little research has investigated how teacher efficacy, burnout and teacher identity intersect in the lives of teachers across their careers. In addition, no work has approached the experiences of teachers using praxis shock as a way to investigate the interrelationships that might exist between self-efficacy, well-being and burnout.

This study examined the perceptions of music teachers specifically, as it has been consistently argued that the experiences of early career music teachers are characterized by practical challenges associated with teaching the discipline area (Krueger, 2001; Scheib, 2006; Ballantyne, 2007; Conway, 2012; Legette, 2013). This is implicated in the findings by Buonomo et al. (2017), who argue that the contextual stressors provided by teaching roles which require extra-curricular responsibilities and workload (like music teachers) are at greater risk of burnout. Much has also been made of the particular nature of music teacher professional identity, whereby performing music (or seeing oneself as a musician), is central to the conception of success as a teacher (Mark, 1998; Ballantyne, 2005, 2007; Ballantyne and Grootenboer, 2012; Ballantyne et al., 2012; Ballantyne and Zhukov, 2017). This work thus extends previous work into the development of productive music teacher identities in pre-service in-service music teachers (Ballantyne, 2007; Ballantyne et al., 2009, 2012; Ballantyne and Zhukov, 2017).

This article explores (1). how teachers’ self-reported levels of burnout and self-efficacy, well-being and praxis shock vary as a function of time in the profession, and (2). the effect of praxis shock and the extent to which it can influence the reported experiences of burnout, well-being and self-efficacy in practicing music teachers. Information was gathered around the nature of music teachers’ experiences across the career span, so as to explore variation in these experiences at different career stages. Specifically, we predicted that reports of praxis shock and burnout would be more common in earlier career teachers and decrease across the career span, while well-being and self-efficacy would have the opposite relationship. Investigating the relationships between these factors create improved understandings around the experiences of music teachers throughout their career. The findings of this paper thus may be used to inform support mechanisms for teachers who may be experiencing difficulties in their jobs, and are at risk of ‘burning out (Day and Gu, 2007).

## Materials and Methods

A questionnaire was designed to explore the experiences of music teachers as they progressed through their career.

### Development of the Questionnaire and Conceptual Frame

The questionnaire comprised scales investigating music teacher self-efficacy and socialization into the profession, specifically looking at praxis shock, well-being, burnout and self-efficacy and how they change over time.

#### Praxis Shock

Measuring the experience of praxis shock provides a primary contribution to previous research in this field. The subscale, developed for the purpose of this study, was based on a review of prior theoretical work (Mark, 1998) and qualitative work (Kelchtermans and Ballet, 2002; Ballantyne, 2007) where praxis shock was associated with early-career teachers’ surprise or shock in relation to aspects of socialisation, workload, isolation, induction and relationships with students and staff. We considered praxis shock to be the feeling of surprise, shock or disequilibrium that results from a discrepancy between an expectation of a workplace environment and what it should be like, and the reality which may be somewhat different. The questions relating specifically to praxis shock measured 1. the degree of shock or surprise associated with these different components of teaching life, and 2. the degree to which there was dissonance between expectations of teaching life and reality experienced (see Appendix A).

#### Burnout

Burnout was examined using items from the Copenhagen Burnout Inventory (CBI) (Kristensen et al., 2005) which defines this construct as “a state of physical, emotional, and mental exhaustion that results from long-term involvement in work situations that are emotionally demanding” (2005, p. 196). The current questionnaire incorporated the CBI items measuring work-related and client-related burnout. CBI items relating to a third domain — personal burnout — were omitted due to redundancy with other questionnaire items. It is notable that the CBI was originally designed to be applicable across a range of work contexts: the “work” in this instance is teaching, and the “clients” are the students. Work-related burnout is defined as the “degree of physical and psychological fatigue and exhaustion that is perceived by the person as related to his/her work”, while client (student)-related burnout is defined as the “degree of physical and psychological fatigue and exhaustion that is perceived by the person as related to his/her work with clients” (Kristensen et al., 2005, p. 197). Importantly, the items are intended to measure a direct attribution of work or clients to one’s state of fatigue, and not another external factor.

Items measuring work-related burnout were divided into two parts. The first three questions related to intensity of burnout (specifically in relation to teaching) and were measured on a 5-point Likert scale. The final four questions probed the frequency of work burnout on a 5-point Likert scale (see Appendix A). The student-related burnout included items explored the extent to which students contributed to teachers’ burnout, and the frequency of the student-related burnout. These were also ranked on a 5-point scale. The full complement of questions referred to in this paper (including division into *student-related burnout* and *work-related burnout*) are found in Appendix A.

#### Well-Being

The questionnaire utilized the World Health Organisation-Five Well-Being Index (1998) version). All items included in this five-item scale are positively worded and designed to measure psychological well-being through such dimensions as mood, vitality and general interest in life^1^ (see Appendix A). It is scored on a 6-point scale, which was reversed for this questionnaire to reflect the same order as the scales in the other questions. The six points range from 0 – At no time, to 5 – All of the time.

#### Self-Efficacy

Self-efficacy was investigated as it related to teaching activities. Teaching self-efficacy is also related to the individual teacher’s estimation of their resilience to changes in circumstances and context. This component of the questionnaire was drawn from the Music Careers Questionnaire II (Hargreaves et al., 2007), although a 6-point Likert scale (strongly disagree to strongly agree) has been utilized. The wording of these items was specifically phrased “in terms of can do rather than will do”, to tap “perceived capability” in accordance with Bandura’s guidelines (2006, p. 308). The 17 statements covered elements of teaching practice such as lesson planning (e.g., “When I plan lessons, I am certain I can make them work”), perseverance (e.g., “If a lesson goes poorly the first time, I try again until it works better”), avoidance (e.g., “I avoid facing difficult situations in my teaching”), determination (e.g., “When I decide to do something, I go right to work on it”) and confidence (e.g., “I feel insecure about my teaching; I am a self-reliant teacher”).

Tschannen-Moran and Woolfolk-Hoy (2001) argue that teachers with greater self-efficacy are more able to cope with change. Thus, self-efficacy was also investigated in terms of respondent’s self-reported ability to “cope with change” and respondents rated confidence in their capacity to demonstrate a range of skills and behaviors relating to coping with change in schooling environments. This component of the questionnaire borrowed from a bank of questions developed by Skaalvik and Skaalvik (2010) in their investigation of teacher self-efficacy and teacher burnout, and is designed to investigate how “certain” participants were about various aspects of teaching. A 7-point Likert scale was used with modification (Skaalvik and Skaalvik did not specify descriptors for 2, 4 and 6 in the Likert scale, so for the purpose of this study, the complete range of descriptors was assumed): 1– Not at all certain, 2 – Very uncertain, 3 – Quite uncertain, 4 – Neither certain or uncertain, 5 – Quite certain, 6 – Very certain, 7 – Absolutely certain. The categories have been used as they were in the original, and are from the cluster of questions related to coping with change. They are: “successfully use any instructional method that the school decides to use”; “manage instruction regardless of how it is organized”; “manage instruction even if the curriculum is changed”, and; “teach well even if you are told to use instructional methods that would not be your choice”. Again, the “can” and “I” wording utilized is reflective of the guides suggested by Bandura (2006). The full complement of questions referred to in this paper (including division into *self-efficacy* and *self-efficacy: cope with change*) are found in Appendix A.

### Reliability

The reliability of all measures was assessed using Cronbach’s alpha. All measures had a reliability greater than 0.8 (see Table 1).

**TABLE 1:** Reliability of measures.

Measure	Cronbach’s alpha	Number of items
Praxis shock	0.886	15
Self-efficacy	0.845	17
Self-efficacy: CwC	0.868	4
Work-related burnout	0.883	7
Student-related burnout	0.841	6
Well-being	0.870	5

### Participants and Procedure

In order to address the research questions, an online questionnaire was distributed to music teachers via an online community of practice, advertisements in professional list-serves, social media, and professional associations. This questionnaire was sent out over 3 years, in 2012, 2013, and 2014.

A total of 1021 respondents attempted the questionnaire with 836 people completing at least 50% of the questionnaire. Participants who completed less than 50% of the questionnaire were removed from all analyses. Because of the ethics requirements associated with voluntary participation in online questionnaires, if someone chose not to answer a question, they were excluded from the analyses that utilize that item. This explains the varied number of responses in each item of the analyses presented.

As Table 2 shows, 81% of respondents were female, with 19% male. The vast majority of respondents (85%) came from Australia, with additional respondents from the United States (9%) and other countries (6.2%). Of the Australian respondents, the majority came from Queensland (45%), followed by New South Wales (20%), Western Australia (16%), and Victoria (11%). The sample had a large number of younger (under 25) and older (51–60) respondents (25% in each instance), with the remaining 50% of respondents aged 26–50. 63% of respondents taught at one school, and 20% taught at two schools, with the majority teaching at State Schools (55%), and 29% teaching at Christian or Catholic Schools. 31% of respondents had completed some sort of dual degree in Music or Arts and Education. 20% had completed straight degrees in Education. 26.2% had completed a Bachelor of Music, and 22.9 had completed a Graduate Diploma of Education. 36.8% had completed another (unnamed) degree.

**TABLE 2:** Demographic information of respondents.

Years teaching^1^ *n* = 702	Age^2^ *n* = 665	Gender *n* = 675	School type *n* = 632	Country *n* = 666	For Australian Respondents, State *n* = 477	At how many schools do you teach? *n* = 456	Course completed *n* = 1063^∗^		Type of teacher *n* = 1328^∗^	
0–1 years = 14.7%	<26 = 25%	Male 19%	Independent, non-Christian school = 6.7%	Australia = 85.1	ACT = 3.4%	1 = 62.7%	Bachelor of Music/Bachelor of Education	24.1%		
2–3 years = 3.7%	26–30 = 14.9%	Female 81%	State School = 55.1%	United States = 8.7%	NSW = 20.3%	2 = 19.5%	Bachelor of Arts/Bachelor of Education	6.9%	Secondary school classroom music teacher	36.8%
4–5 years = 10.3%	31–40 = 18.3%		Catholic School = 12.8%	Other = 6.2	NT = 0.6%	3 = 7.9%	Bachelor of Education (Secondary)	5.9%	Primary school classroom music teacher	48.1%
6–10 years = 14.4%	41–50 = 19.6%		Church/Christian School (other than Catholic) = 13.8%		QLD = 45.3	4 = 2.6%	Bachelor of Education (Primary)	14.1%	Instrumental music teacher (Secondary)	33.6%
11–15 years = 10.0%	51–60 = 24.5%		Other = 11.7%		SA = 2.3%	5 = 3.7%	Bachelor of Music	26.2%	Instrumental music teacher (Primary)	24.3%
16–20 years = 10.1%					TAS = 1.7%	6 = 1.5%	Bachelor of Arts	11.3%	Studio music teacher	39.7%
More than 20 years teaching = 36.9%					VIC = 10.7%	7 or more = 2%	Graduate Diploma of Education	22.9%	Other (please describe)	15.2%
					WA = 15.7%		Graduate Diploma of Music	6.5%		
							Master of Teaching	5.3%		
							Other (please list as many as required)	36.8%		

*^∗^Values exceed the total sample size as respondents could provide multiple responses to many of these questions. ^1^It should be noted that responses were coded at ‘21’ years of teaching when respondents selected ‘more than 20 years teaching’ in the first year of the questionnaire (2012). Although the questionnaire was initially designed for early-career teachers, when it became apparent that this questionnaire was applicable and interesting to more experienced teachers, subsequent iterations asked respondents a series of questions related to years in the profession, to more accurately assess their number of years teaching. Respondents were asked how long they had been teaching, in which year they began teaching, and the year of their graduation from university. In some cases, respondent’s age did not match with the number of years that they said they had been teaching, and the years since they graduated university. This is because of the prevalence of private tuition as a source of income during university for music students. However, for the purposes of this study, we considered the number of years teaching to be the number of years teaching since graduation, and therefore adjusted the number of years teaching variable to reflect this. ^2^When the data from 2012 was being cleaned, there was the need to assign an actual value for those who were outside of the range option provided for ‘age.’ It was deemed important to include those people in the sample (those who had provided an age of ‘less than 20 years,’ and those who had provided an age of ‘more than 50 years’), and they were given a nominal age of ‘19’ and ‘51’ respectively. Those respondents who chose not to reveal their age were removed from the ‘age’ sample. This is standard practice in the cleaning of data. This ensured that the merging of the data from 2012, 2013 and 2014 was consistent.*

### Missing Data

The data is not as precise for the latter period of the career. In the initial iteration of the questionnaire (2012), respondents who selected “more than 20 years teaching” were given a code of “21” for “years of teaching”. In subsequent iterations (2013 and 2014), additional questions associated with how long they had been teaching, in which year they began teaching and the year of their graduation from university. For analysis purposes, cases of years-teaching greater than 21 were reclassified as 21 years-teaching. That is, 21 years was the upper limit on the years teaching scale.

## Results and Discussion

### How Teachers’ Self-Reported Levels of Burnout and Self-Efficacy, Well-Being and Praxis Shock Vary Over Time in the Profession

Regression analyses were used to evaluate the degree to which the constructs of interest (e.g., praxis shock, self-efficacy, well-being, and burnout) varied as a function of time spent teaching in years. Participants’ means score on each measure were used in the regression analyses. All analyses were run using Stata (15) software.

**FIGURE 1:**
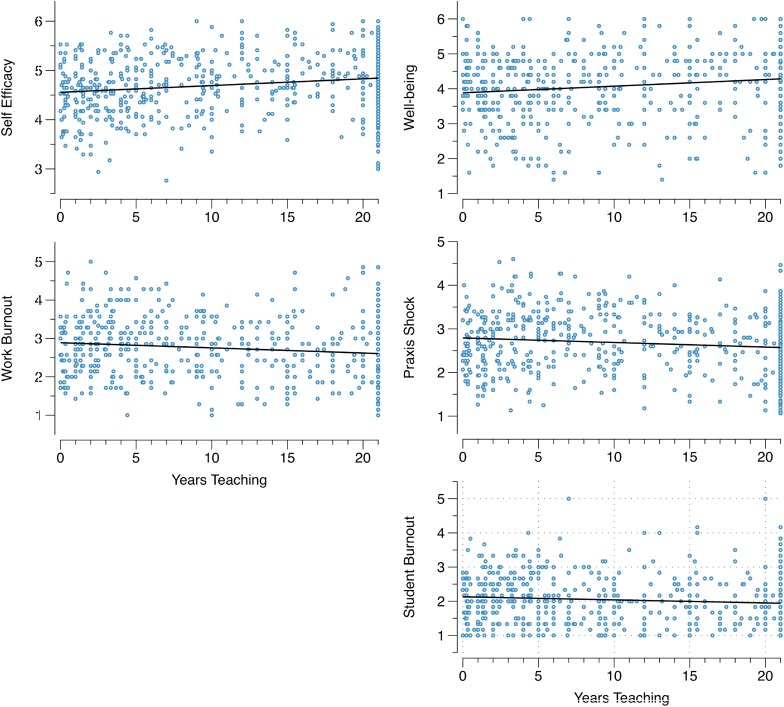
Self-efficacy, well-being, work burnout, praxis shock, and student burnout as a function of years teaching, all with linear fits to the data.

**FIGURE 2:**
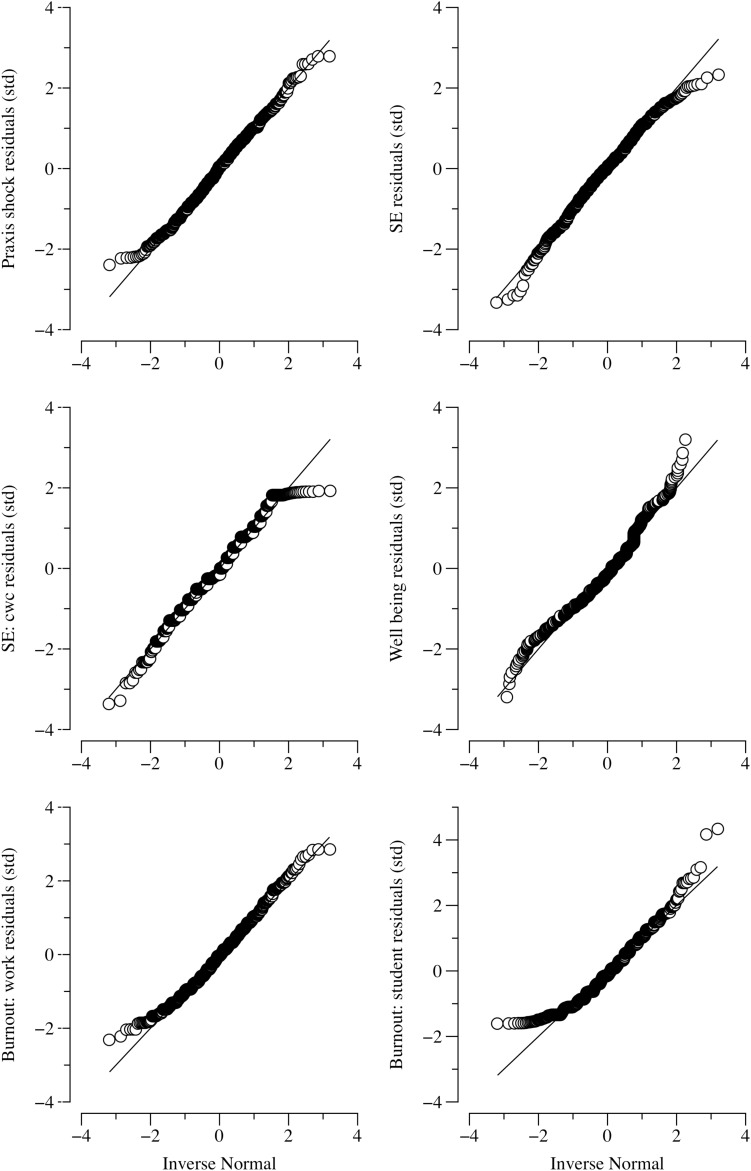
Quantile-quantile plots showing normality fits for all linear models.

Linear regression analyses revealed significant (*p* < 0.05) linear relationships between years teaching and all the outcome variables except for *self-efficacy: cope with change*. Consistent with our predictions, *self-efficacy* and *well-being* were positively related to years teaching, while *work-related burnout*, *student-related burnout*, and *praxis shock* were negatively related to years teaching (see Table 3). Figure 1 provides graphical representations of the linear models described here. Regarding the assumptions of the linear models, in all cases, the error variance was constant as a function of years teaching. That is, the assumption of homoscedasticity was met. Normality was assessed using quantile-quantile plots (see Figure 2). Both *self-efficacy: cope with change* and *student-related burnout* show some divergence from normality, though only in the tails. Neither a natural log or square root transformation could correct this. Otherwise, the model residuals appear normally distributed.

**TABLE 3:** Change in psychological outcomes as a function of years teaching.

DV	Linear R^2^ (adjusted)	β	*F*	*p*
Self-efficacy	0.036 (0.035)	0.014	28.73	<0.001
Self-efficacy: CwC	0.016 (0.003)	0.005	1.20	0.274
Well-being	0.026 (0.025)	0.019	19.11	<0.001
Work-related burnout	0.018 (0.017)	−0.013	13.54	<0.001
Student-related burnout	0.010 (0.009)	−0.009	8.03	0.006
Praxis shock	0.015 (0.014)	−0.011	10.82	0.001

Early career respondents in our sample tended to report increased levels of praxis shock which declined steadily with time spent within the profession. However, it is important to note that this linear decline was small, and it is clear that praxis shock remained an issue beyond the early years in the profession. Indeed, there are clear cases of respondents who have taught for 21 years or more experiencing high levels of praxis shock. This is an interesting phenomenon that was not anticipated in this study and is not previously documented. Persistence of praxis shock may be evident for a number of reasons, but is likely to reflect the fact that praxis shock can emerge when there is a discrepancy between the expectation of teaching life, and the realities faced by teachers. That this was a reality for experienced teachers as well as early career teachers, was unexpected. Praxis shock amongst experienced teachers may reflect the distance between the changing philosophies and expectations held by statutory bodies and management, and which teachers are expected to embody. Or perhaps music teachers commonly find themselves in new (more senior) roles and with new duties, irrespective of teaching experience. Any dissonance between their perceived skill set and the skills required may prompt praxis shock and burnout. Indeed, the praxis shock observed in this data set may be related to recent changes in terms of requirements, and the changes in relation to “what teaching is all about” in many schools. The current data do not shed light on this, but certainly provides the impetus for further investigation and at best may indicate an unexpected need for support for this demographic of the profession.

In summary, the series of linear regressions reported above show that teachers’ self-reported levels of burnout, self-efficacy, well-being, and praxis shock vary over their time in the teaching profession. More specifically, we found that *self-efficacy* and *well-being* showed a small steady increase with the number of years teaching. While *praxis shock*, *work, and student-related burnout* behaved in the opposite manner, where both decreased slightly with number of years teaching.

### How the Constructs of Well-Being, Burnout and Self-Efficacy Relate to Teachers’ Experiences of Praxis Shock

The similarities observed between the linear models above suggest that there may be some degree of interrelationship between praxis shock and the other psychological constructs. The working hypothesis was that experience with praxis shock may account for differences in well-being, student- and work-burnout and self-efficacy across the teaching career. While it has been argued that the experience of praxis shock can result in burnout (Ballantyne, 2007; Ballantyne and Zhukov, 2017), and is also associated with attrition and reduced job satisfaction (Kelchtermans and Ballet, 2002; Ballantyne, 2005, 2007; Hong, 2010; Skaalvik and Skaalvik, 2010), existing literature is restricted to qualitative documentation of the existence of praxis shock in teachers (Stokking et al., 2003; Smagorinsky et al., 2004; Ballantyne, 2007), indicating that it might be linked to well-being, student- and work-related burnout, and self-efficacy. As such, we ran a series of mediation analyses to test whether praxis shock might mediate the relationship between years of teaching and self-efficacy, well-being, and student- and work-related burnout.

#### Mediation Analyses

Mediation analyses were run to test whether the relationship between years in the profession and all other factors was mediated by praxis shock. In all cases, the relationship was significant (see Table 4), with praxis shock *partially* mediating the direction of the outcomes over time. The one exception was student-related burnout where we observed a *full* mediation of the relationship.

**TABLE 4:** Statistical output for the mediation tests investigating praxis shock as a mediator of the relationship between years teaching and significant outcomes.

	*Mediation* β (*p*)	*Bootstrap CI 95%*
*Well-being*	0.0033 (0.049)	0.0004 to 0.0061
*Self-efficacy*	0.0012 (0.038)	0.00004 to 0.0023
*Work-related burnout*	−0.0039 (0.033)	−0.007 to −0.0005
*Student-related burnout*	−0.0026 (0.025)	−0.005 to −0.003

The mediation analyses provide moderate evidence that praxis shock is a contributing factor to the relationship between years teaching and our outcomes variables *self-efficacy, well-being* and *work-* and *student-related burnout.* That is, as the degree of praxis shock reduces as teachers stay in the profession, this reduction partially mediates the relationship between increased years of teaching experience, and the outcomes of *burnout, self-efficacy*, and *well-being*. Higher levels of *praxis shock* contribute to increased levels of *burnout* and decreased levels of *well-being* and *self-efficacy* and vice versa. In characterizing the degree of evidence as moderate, we are acknowledging that the mediation analyses are close to the significance threshold. Although scientific conclusions should not be based on whether *p*-values passes a specific threshold, future studies may seek to replicate these effects to confirm their reliability.

#### Exploratory Analyses

Finally, we ran one last set of exploratory analyses. It’s clear from Figure 1 that a large amount of variance in the data is unaccounted for by the simple linear models. That is, the relationship between praxis shock and years teaching appears more nuanced than a simple linear one. Certainly, from a theoretical perspective, we might expect the relationships to vary between early- versus late-career teachers. Most of the previous literature has focussed on the existence of praxis shock in the early years of the profession, and in particular the experiences of early career music teachers (Ballantyne, 2007; Ballantyne and Zhukov, 2017). This is logically because it focusses on the discrepancies between university preparation and expectations prior to graduation, and the realities of teaching. To explore possible subtle differences in the relationships explored above between the early and late career teacher cohorts, we split the data according to early career (0–5 years) and more advanced career teachers (>5 years). A series of Pearson’s correlations between years teaching and our psychological constructs of interest were run for earlier (≤5) and later career teachers (>5). We stress that this set of correlation analyses are very much exploratory, run in response to the complex pattern of data observed above. The results are reported in Table 5.

**TABLE 5:** Bivariate relationships between number of years teaching and all outcomes for early-career, mid-career, and late-career periods in the profession.

	Well-being		Self-efficacy		Self-efficacy CWC		Work burnout		Student burnout		Praxis shock	
						
	*r*	*p*	*r*	*p*	*r*	*p*	*r*	*p*	*r*	*p*	*r*	*p*
<5 year (early-career)	−0.175	0.013	0.080	0.259	−0.024	0.732	0.165	0.020	0.145	0.041	0.184	0.010
5–21 year (mid-career)	0.141	0.001	0.050	0.239	−0.042	0.328	−0.117	0.007	−0.065	0.144	−0.222	<0.001

The key result from Table 5 is that, in some cases, the relationship between years teaching and the respective psychological construct appears to differ as a function of early versus later career. Specifically, for *well-being*, *work burnout*, and *praxis shock*, the direction of the relationship for early and later career teachers is reversed. *Well-being* looks to decrease very slightly in the first 5 years of teaching and increase thereafter. The opposite is true for *praxis shock* and *work burnout*. No effect of *self-efficacy* or *self-efficacy*: cope with change was observed here. It appears that early-career teachers are most vulnerable to experiencing *praxis shock* and *burnout*. However, increased time (beyond ∼5 years) in the profession may bring only a small reprieve from these experiences.

## Conclusion

This paper explored the complexities that lie within the relationships between self-reported levels of *self-efficacy, praxis shock, burnout*, and *well-being* for music teachers, and time in a career. This study provides evidence that levels of *burnout, well-being, self-efficacy*, and most importantly *praxis shock*, are influenced by the number of years that teachers have been teaching. Additionally, levels of *praxis shock* predict levels of *burnout*, *well-being* and *self-efficacy*.

Our findings demonstrate that there are distinct challenges in the early career and late career periods of a music teacher’s working life. Day and Gu (2007) found that teachers who were 24–30 years post-graduation found themselves in a phase where they have to adjust to changes associated with “deteriorating pupil behaviors, adverse personal events, resentment at ‘being forced to jump through hoops by a constant stream of new initiatives, and career stagnation”. Teachers in this life phase are at greater risk of being less effective, largely due to the likelihood of experiencing “extreme professional life phase scenarios (p. 437). It is highly likely that the experiences both early in the career and later in the career are associated with attrition from the profession. At the very least, providers of pre-service teacher education and in-service teacher education would be well advised to consider the impact of praxis shock across the career in terms of burnout and well-being.

As praxis shock is largely about the dissonance between expectations and the realities of working life, it is likely that awareness of the trends identified in this study will enhance awareness of the support necessary for teachers across the career - where praxis shock appears to be evident even later into the career. Targeted career-stage support needs to be developed for teachers, to assist them in navigating difficult periods successfully. Perhaps the development of a “proactive praxis” strategy in in-service teacher support will enable the pre-empting of the likelihood of praxis shock. For example, Gu and Day’s (2007) work examining teacher identities and life stages suggest that in-service support focussing on ways to address external policy initiatives, helped more experienced teachers maintain enthusiasm and commitment to teaching during periods of praxis shock. Another suggestion is the use of strategies from positive psychology - qualitative findings with early-career teachers suggest that resilience developed through a positive psychology approach is indicated as protective against the negative impacts of praxis shock (Ballantyne and Zhukov, 2017). It is likely that support designed for experienced teachers might initially address similar issues to those addressed early in the career, but further research is warranted to investigate the nature of later-career praxis shock, in order for a nuanced support approach to be developed.

Support strategies for pre-service teachers are equally important. Demystifying the teaching experience and actively encouraging pre-service teachers to engage critically with their teaching experiences is likely to be key to negating praxis shock. Support strategies might involve addressing well-being and self-efficacy explicitly during university education, so that prior to embarking on their career, pre-service teachers might be better equipped to navigate successfully through the difficult first five years, minimizing their likelihood of experiencing burnout.

As identified by Hong (2010), emotional burnout is critical in influencing success in the profession. The current study goes further by showing that reported praxis shock impacts on burnout. It should be noted that the findings by Fiorilli et al. (2017) indicate that teachers who may be undergoing the experience of praxis shock may interpret students’ actions in a more negative way - this may be associated with student-related burnout.

The links found between praxis shock and self-efficacy echo the arguments put forward by Gibbs (2001), who claimed that self-efficacy is the key to managing anxiety and stress associated with praxis shock. Gibbs emphasized that strategies specifically aimed at supporting teachers should result in enhanced self-efficacy.

This study also raises questions about other similar professions where burnout has been identified at the beginning of the career (for example, in health professions, psychology, law enforcement, legal, and even academia). Indeed, as this paper looked exclusively at teachers identifying as music specialists, it would be interesting to see to what extent this trend is evident in teachers with other specializations, and even generalist primary teachers. The praxis shock questionnaire items developed for the current study provide a useful tool for the investigation of other professions.

Our data do not reveal whether some of the issues have to do with time in the teaching role or perhaps changes to the nature of teaching over time. To address this question, a longitudinal study is required. The changing nature of schools, teaching methods, accountability demands, and technology infiltrating the classroom may damage self-efficacy for teachers who have been in the industry for many years (>5), and this may be the reason for persistent praxis shock in the later parts of the career. In addition, the varied contexts from which the respondents came (including across different countries), means that contextualization in terms of common experiences is not possible. Indeed, even if the data were limited to one country, common experiences are unlikely to be evident; even within Australia, the experiences of music teachers vary from school to school. Future analyses of the longitudinal data (the 2nd and 3rd phases of questionnaire analysis with the same respondents) are likely to shed more light on how the experience of praxis shock and burnout, as well as teachers’ well-being and self-efficacy change over time.

It should also be noted that this data was collected from teachers who were currently teaching, and it therefore, does not include those teachers who had left the profession. Hong (2010) found that those teachers who did burn out and consequently left teaching were more likely to admit to being extremely stressed about the work itself (work burnout in this study). Also evident in Hong’s research is the impact of student-related stress (p. 1537). Although teachers who remained in the profession reported less emotional burnout, it did not seem to affect their professional identities and lives (Hong, 2010).

The apparent occurrence of praxis shock throughout the career is likely to have an impact on the development of sustainable and productive professional identities. In the case of music teachers, it has been argued that how music teachers think about themselves as musicians and as educators, and how proficient they are in these areas (their self-efficacy), is demonstrably associated with their identities (Hargreaves et al., 2007; Ballantyne and Grootenboer, 2012; Ballantyne et al., 2012; Ballantyne and Zhukov, 2017). Professional identity development has consistently been related to self-efficacy (Hargreaves et al., 2007) and teacher identity arguably has a prominent role to play in teacher praxis (Beijaard et al., 2004; Sachs, 2005; Diez and Raths, 2007). More needs to be known about how professional contexts, praxis shock and identity interrelate, and how these factors over the career play a role in the development of confidence and effectiveness as a professional. Investigating the nuances in these interrelationships is an area for future research and one that will hopefully emerge from the analysis of the fuller data from this project.

## Data Availability Statement

The datasets generated for this study will not be made publicly available as it was a condition of ethical approval that only the authors are able to access the data.

## Ethics Statement

The studies involving human participants were reviewed and approved by the University of Queensland Ethics Committee. Participants read a full information statement about the study, and gave their consent to participate prior to beginning the questionnaire. The participants provided their written informed consent to participate in this study.

## Author Contributions

JB led all aspects of this project and writing of the manuscript. JR assisted with the statistical analysis.

## Conflict of Interest

The authors declare that the research was conducted in the absence of any commercial or financial relationships that could be construed as a potential conflict of interest.

## Footnotes

1 http://www.who-5.org

## Appendix

**TABLE:** Questionnaire items.

Scale and item number	Item text
*The Self-Efficacy Scale 1*	When I plan lessons, I am certain I can make them work
*The Self-Efficacy Scale 2*	One of my problems is that I cannot get down to lesson preparation when I should
*The Self-Efficacy Scale 3*	If a lesson goes poorly the first time, I try again until it works better
*The Self-Efficacy Scale 4*	When I set important goals for my teaching, I rarely achieve them
*The Self-Efficacy Scale 5*	I give up on things before completing them
*The Self-Efficacy Scale 6*	I avoid facing difficult situations in my teaching
*The Self-Efficacy Scale 7*	If something on the syllabus appears complicated, I will not bother trying to teach it
*The Self-Efficacy Scale 8*	When I have something unpleasant to do, I stick to it until I finish it
*The Self-Efficacy Scale 9*	When I decide to do something, I go right to work on it
*The Self-Efficacy Scale 10*	When trying something new in my teaching, I soon give up if I am not initially successful
*The Self-Efficacy Scale 11*	If something unexpected happens during a lesson, I do not handle it well
*The Self-Efficacy Scale 12*	I avoid trying something new in my teaching if it looks too difficult for me
*The Self-Efficacy Scale 13*	Failure just makes me try harder
*The Self-Efficacy Scale 14*	I feel insecure about my teaching
*The Self-Efficacy Scale 15*	I am a self-reliant learner
*The Self-Efficacy Scale 16*	I give up easily
*The Self-Efficacy Scale 17*	I do not seem capable of dealing with most problems that come up in my teaching activities
*Teacher Self-Efficacy Cope with Change 1*	How certain are you that you can successfully use any instructional method that the school decides to use?
*Teacher Self-Efficacy Cope with Change 2*	How certain are you that you can manage instruction regardless of how it is organized (group, composition, mixed age groups, etc.)?
*Teacher Self-Efficacy Cope with Change 3*	How certain are you that you can manage instruction even if the curriculum is changed?
*Teacher Self-Efficacy Cope with Change 4*	How certain are you that you can teach well even if you are told to use instructional methods that would not be your choice?
*World Health Org Well-being Questionnaire 1*	Over the last two weeks I have felt cheerful and in good spirits
*World Health Org Well-being Questionnaire 2*	Over the last two weeks I have felt calm and relaxed
*World Health Org Well-being Questionnaire 3*	Over the last two weeks I have felt active and vigorous
*World Health Org Well-being Questionnaire 4*	Over the last two weeks I woke up feeling fresh and rested
*World Health Org Well-being Questionnaire 5*	Over the last two weeks my daily life has been filled with things that interest me
*Copenhagen Burnout Inventory/Work-Related 1*	Is teaching emotionally exhausting?
*Copenhagen Burnout Inventory/Work-Related 2*	Do you feel burnt out because of teaching?
*Copenhagen Burnout Inventory/Work-Related 3*	Does teaching frustrate you?
*Copenhagen Burnout Inventory/Work-Related 4*	Do you feel worn out at the end of the working day?
*Copenhagen Burnout Inventory/Work-Related 5*	Are you exhausted in the morning at the thought of another day at work?
*Copenhagen Burnout Inventory/Work-Related 6*	Do you feel that every working hour is tiring for you?
*Copenhagen Burnout Inventory/Work-Related 7*	Do you have enough energy for family and friends during leisure time?
*Copenhagen Burnout Inventory/Student-Related 1*	Do you find it hard to work with students?
*Copenhagen Burnout Inventory/Student-Related 2*	Do you find it frustrating to work with students?
*Copenhagen Burnout Inventory/Student-Related 3*	Does it drain your energy to work with students?
*Copenhagen Burnout Inventory/Student-Related 4*	Do you feel that you give more than you get back when you work with students?
*Copenhagen Burnout Inventory/Student-Related 5*	Are you tired of working with students?
*Copenhagen Burnout Inventory/Student-Related 6*	Do you sometimes wonder how long you will be able to continue working with students?
*Praxis Shock Inventory Part A 1*	When you first began teaching (work), were you **surprised** or even **shocked** with any aspects of the workload and expectations placed on you?
*Praxis Shock Inventory Part A 2*	When you first began teaching (your job), were you **surprised** or even **shocked** with any aspects of the school culture?
*Praxis Shock Inventory Part A 3*	When you first began teaching (your job), were you **surprised** or even **shocked** with any aspects of the time and energy required to meet the demands of your job?
*Praxis Shock Inventory Part A 4*	When you first began teaching (your job), were you **surprised** or even **shocked** with any aspects of the level of support you received?
*Praxis Shock Inventory Part A 5*	When you first began teaching (your job), were you **surprised** or even **shocked** with any aspects of the attitudes or behavior of students?
*Praxis Shock Inventory Part A 6*	When you first began teaching (your job), were you **surprised** or even **shocked** with any aspects of your relationship with other staff?
*Praxis Shock Inventory Part B 1*	How accurate were your pre-service ideas about day-to-day life as a teacher?
*Praxis Shock Inventory Part B 2*	To what extent do the pressures of the job impact on your ability to be the teacher you would hope to be?
*Praxis Shock Inventory Part B 3*	Were any of your pre-service beliefs and ideas about teaching inaccurate?
*Praxis Shock Inventory Part B 4*	Are other teachers and staff as supportive as you thought they would be?
*Praxis Shock Inventory Part B 5*	Has daily life as a teacher challenged any of the pre-service beliefs and ideas about teaching you held about life working as a teacher?
*Praxis Shock Inventory Part B 6*	How realistic were the pre-service expectations you held about life as a teacher?
*Praxis Shock Inventory Part B 7*	Do you think you began your career with a good grasp of what school life is like for a teacher?
*Praxis Shock Inventory Part B 8*	How closely do your preconceptions about being a teacher fit with the realities of teaching?
*Praxis Shock Inventory Part B 9*	Have you experienced any clash between the vision of teaching you had before beginning and the realities of the job?

## References

[B1] Ballantyne J. (2005). “Identities of music teachers: implications for teacher education,” in *Proceedings of the Teacher Education: Local and Global: Australian Teacher Education Association Conference*, ed. Cooper M. (Gold Coast, QLD: Australian Teacher Education Association), 39–44.

[B2] Ballantyne J. (2007). Documenting praxis shock in early-career australian music teachers: the impact of pre-service teacher education. *Int. J. Music Educ.* 25 181–191. 10.1177/0255761407083573

[B3] Ballantyne J. Grootenboer P. (2012). Exploring relationships between teacher identities and disciplinarity. *Int. J. Music Educ.* 30 368–381. 10.1177/0255761412459165

[B4] Ballantyne J. Harrison S. Barrett M. Temmerman N. (2009). *Bridging Gaps in Music Teacher Education: Developing Exemplary Practice Models Using Peer Collaboration.* Sydney, NSW: Australian Learning and Teaching Council.

[B5] Ballantyne J. Kerchner J. Aróstegui J. (2012). Developing music teacher identities: an international multi-site study. *Int. J. Music Educ.* 30 211–226. 10.1177/0255761411433720

[B6] Ballantyne J. Zhukov K. (2017). A good news story: early-career music teachers’ accounts of their “flourishing” professional identities. *Teach. Teach. Educ.* 68 241–251. 10.1016/j.tate.2017.08.009

[B7] Bandura A. (2006). Toward a psychology of human agency. *Perspect. Psychol. Sci.* 1 164–180. 10.1111/j.1745-6916.2006.00011.x 26151469

[B8] Beauchamp C. Thomas L. (2009). Understanding teacher identity: an overview of issues in the literature and implications for teacher education. *Cambridge J. Educ.* 39 175–189. 10.1080/03057640902902252

[B9] Beijaard D. Meijer P. Verloop N. (2004). Reconsidering research on teachers’ professional identity. *Teach. Teac. Educ.* 20 107–128. 10.1016/j.tate.2003.07.001

[B10] Betoret F. (2006). Stressors, self-efficacy, coping resources, and burnout among secondary school teachers in Spain. *Educ. Psychol.* 26 519–539. 10.1080/01443410500342492

[B11] Buonomo I. Fatigante M. Fiorilli C. (2017). Teachers’ burnout profile: risk and protective factors. *Open Psychol. J.* 10 190–201. 10.13075/ijomeh.1896.00238 26159952

[B12] Conway C. (2012). Ten years later, experienced teacher reflections on “beginning music teacher perceptions of district-sponsored induction programs” (2001). *Bull. Council Res. Music Educ.* 193 63–76.

[B13] Day C. Gu Q. (2007). Variations in the conditions for teachers’ professional learning and development: sustaining commitment and effectiveness over a career. *Oxf. Rev. Educ.* 33 423–443. 10.1080/03054980701450746

[B14] Day C. Kington A. (2008). Identity, well-being and effectiveness: the emotional contexts of teaching. *Pedagogy Cult. Soc.* 16 7–23. 10.1080/14681360701877743

[B15] Diez M. E. Raths J. D. (2007). *Dispositions in Teacher Education.* Charlotte, NC: Information Age Pub.

[B16] Fernet C. Guay F. Senécal C. Austin S. (2012). Predicting intraindividual changes in teacher burnout: the role of perceived school environment and motivational factors. *Teach. Teach. Educ.* 28 514–525. 10.1016/j.tate.2011.11.013

[B17] Fiorilli C. Pepe A. Buonomo I. Albanese O. (2017). At-risk teachers: the association between burnout levels and emotional appraisal processes. *Open Psychol. J.* 10 129–139.

[B18] Flores M. Day C. (2006). Contexts which shape and reshape new teachers’ identities: a multi-perspective study. *Teach. Teach. Educ.* 22 219–232. 10.1016/j.tate.2005.09.002

[B19] Gibbs C. (2001). Explaining effective teaching: self-efficacy and thought control of action. *J. Educ. Enquiry* 4 1–14.

[B20] Gu Q. Day C. (2007). Teachers resilience: a necessary condition for effectiveness. *Teach. Teach. Educ.* 23 1302–1316. 10.1016/j.tate.2006.06.006

[B21] Hargreaves D. J. Purves R. M. Welch G. F. Marshall N. A. (2007). Developing identities and attitudes in musicians and classroom music teachers. *Br. J. Educ. Psychol.* 77 665–682. 10.1348/000709906x154676 17908381

[B22] Hong J. Y. (2010). Pre-service and beginning teachers’ professional identity and its relation to dropping out of the profession. *Teach. Teach. Educ.* 26 1530–1543. 10.1016/j.tate.2010.06.003

[B23] Hultell D. Melin B. Gustavsson J. P. (2013). Getting personal with teacher burnout: a longitudinal study on the development of burnout using a person-based approach. *Teach. Teach. Educ.* 32 75–86.

[B24] Kelchtermans G. Ballet K. (2002). The micropolitics of teacher induction. A narrative-biographical study on teacher socialisation. *Teach. Teach. Educ.* 18 105–120. 10.1016/s0742-051x(01)00053-1

[B25] Kristensen T. Borritz M. Villadsen E. Christensen K. (2005). The copenhagen burnout inventory: a new tool for the assessment of burnout. *Work Stress* 19 192–207. 10.1080/02678370500297720

[B26] Krueger P. J. (2001). Reflections of beginning music teachers: the concerns of new teachers frequently overlap, and being aware of these issues may benefit veteran teachers who want to help. *Music Educ. J.* 88 51–54. 10.2307/3399759

[B27] Legette R. M. (2013). Perceptions of early-career school music teachers regarding their preservice preparation. *Update* 32 12–17. 10.1177/8755123313502342

[B28] Lindqvist P. Nordänger U. Carlsson R. (2014). Teacher attrition the first five years – a multifaced image. *Teach. Teach. Educ.* 40 94–103. 10.1016/j.tate.2014.02.005

[B29] Litano M. Major D. (2016). Facilitating a whole-life approach to career development: the role of organizational leadership. *J. Career Dev.* 43 52–65. 10.1177/0894845315569303

[B30] Mark D. (1998). The music teachers’ dilemma – musician or teacher? *Int. J. Music Educ.* 32 3–23. 10.1177/025576149803200102

[B31] Parliament of Australia Media Release (2019). *Parliament Learning From Teachers.* Available at: https://www.aph.gov.au/Parliamentary_Business/Committees/House/Employment_Education_and_Training/TeachingProfession/Media_Releases (accessed November 11, 2019).

[B32] Pillen M. T. den Brok P. J. Beijaard D. (2013). Profiles and change in beginning teachers’ professional identity tensions. *Teach. Teach. Educ.* 34 86–97. 10.1016/j.tate.2013.04.003

[B33] Sachs J. (2005). “Teacher education and the development of professional identity: learning to be a teacher,” in *Connecting Policy and Practice: Challenges for Teaching and Learning in Schools and Universities*, eds Denicolo P. Kompf M. (Oxford: Routledge), 5–21.

[B34] Schaefer L. (2013). Beginning teacher attrition: a question of identity making and identity shifting. *Teach. Teach.* 19 260–274. 10.1080/13540602.2012.754159

[B35] Scheib J. (2006). Policy implications for teacher retention: meeting the needs of the dual identities of arts educators. *Arts Educ. Policy Rev.* 107 5–10. 10.3200/aepr.107.6.5-10

[B36] Shaw J. (2016). Alleviating praxis shock: induction policy and programming for urban music educators. *Arts Educ. Policy Rev.* 119 1–11. 10.1080/10632913.2016.1185655

[B37] Skaalvik E. Skaalvik S. (2007). Dimensions of teacher self-efficacy and relations with strain factors, perceived collective teacher efficacy, and teacher burnout. *J. Educ. Psychol.* 99 611–625. 10.1037/0022-0663.99.3.611

[B38] Skaalvik E. Skaalvik S. (2010). Teacher self-efficacy and teacher burnout: a study of relations. *Teach. Teach. Educ.* 26 1059–1069. 10.1016/j.tate.2009.11.001 24765710

[B39] Skaalvik E. Skaalvik S. (2014). Teacher self-efficacy and perceived autonomy: relations with teacher engagement, job satisfaction, and emotional exhaustion. *Psychol. Rep.* 114 68–77. 10.2466/14.02.pr0.114k14w0 24765710

[B40] Smagorinsky P. Cook L. S. Moore C. Jackson A. Y. Fry P. G. (2004). Tensions in learning to teach: accommodation and the development of a teaching identity. *J. Teach. Educ.* 55 8–24. 10.1177/0022487103260067

[B41] Stokking K. Leenders F. De Jong J. Van Tartwijk J. (2003). From student to teacher: reducing practice shock and early dropout in the teaching profession. *Eur. J. Teach. Educ.* 26 329–350. 10.1080/0261976032000128175

[B42] Tschannen-Moran M. Woolfolk-Hoy A. (2001). Teacher efficacy: capturing an elusive concept. *Teach. Teach. Educ.* 17 783–805. 10.1016/s0742-051x(01)00036-1

[B43] World Health Organisation-Five Well-Being Index (1998). *World Health Organisation-Five Well-Being Index.* Available at http://www.who-5.org (accessed April 17, 2012).

[B44] Zee M. Koomen H. (2016). Teacher self-efficacy and its effects on classroom processes, student academic adjustment, and teacher well-being: a synthesis of 40 years of research. *Rev. Educ. Res.* 84 981–1015. 10.3102/0034654315626801

